# Residents' perspectives on their training in managing errors in health care: an explorative qualitative study

**DOI:** 10.5116/ijme.68d1.1e73

**Published:** 2025-09-29

**Authors:** Bente V. Malling, Mads Skipper, Signe S. Matthiesen, Randsbæk Flemming, Linda M.K. Nielsen, Maja B. Hansen, Jane E. Møller

**Affiliations:** 1 Department of Clinical Medicine, Aarhus University, Denmark; 2 Postgraduate Medical Education, Educational Region North, Denmark; 3 Postgraduate Medical Education, Department of Clinical Medicine, Aarhus University, Denmark; 4 Department of Psychosis, Aarhus University Hospital, Denmark

**Keywords:** Managing medical error, post-graduate training, residents, qualitative

## Abstract

**Objectives:**

This study investigated the perspectives of residents on training in error managing. The research question was: how do residents perceive and experience their training in handling errors in clinical practice?

**Methods:**

The study used a qualitative exploratory design to gain insight into the residents' experiences. The data consisted in seven virtual focus groups with 22 Danish residents from 11 specialties at various educational levels, i.e. from first to fourth year of their training. The data were transcribed and analyzed using reflexive thematic analysis.

**Results:**

The analysis showed three themes: 1) formal education, 2) culture and clinical context, and 3) the need for more training. The residents reported learning primarily about the legal aspects of errors, complaints, and the adverse events reporting system. They emphasized the need for practical training in error disclosure and managing emotional reactions to errors. Training in error managing was described as sporadic in specialist training, often contingent on departmental culture and individual supervisors.

**Conclusion:**

The study revealed that residents perceive their training in error handling as inadequate, particularly in terms of disclosure, emotional reactions, and victim support, and identified the need for greater emphasis on these aspects in both undergraduate education and postgraduate training. The Danish framework for physician roles and specialist training curricula should be revised and a more open culture regarding medical errors fostered. Furthermore, supervisor training is crucial as training in error management should be integrated into the clinical setting where errors occur.

## Introduction

This study explores how residents experience their training in managing errors in clinical practice. Despite efforts to ensure patient safety in health care and healthcare providers’ desire to “do no harm,” errors occur regularly in clinical practice.^1^ The immense human and economic consequences of errors in health care make it imperative for healthcare providers, e.g. doctors to manage errors professionally.^1^^-^^3^ In an effort to reduce errors, adverse events reporting systems have been implemented worldwide to identify, investigate and, if possible, remove systemic causes of errors.^4^^-^^7^ However, even if all systemic causes to errors were eliminated, individual causes of errors would still be present due to what has been called the “human factor”,^2^ and parallel with the implementation of adverse events reporting systems, a movement from a “blame and shame” culture to an approach focused on learning from errors has been evident.^2^^,^^3^ Research has described human reactions to committing medical errors together with the need to support the following three types of victims: first (patients and relatives), second (involved healthcare providers) and third (organizations).^6^^,^^8^^-^^11^

Although individual causes of errors in health care can never be completely eliminated, factors like lack of knowledge and skills can be addressed through education and training.^12^^,^^13^ Furthermore, culture and leadership that enhance learning opportunities for whole departments play an important role in creating openness about errors.^14^ As all doctors are inevitably involved in errors,^2^^,^^3^ this raises the question of how they learn to manage them. The UK's description of Good Medical Practice states that all doctors must be able to manage errors professionally, e.g. be open and honest with patients and relatives when things go wrong.^15^ In line with this, training programs have been suggested for healthcare personnel such as doctors on disclosure and managing one’s own and other healthcare providers’ reactions to errors.^8^^,^^10^^,^^11^^,^^14^^,^^16^^,^^17^ However, recent studies emphasize that junior doctors face challenges in conceptualizing errors and lack structured support mechanisms during and after adverse events or errors.^18^^,^^19^ It has further been demonstrated that residents express a need for more formal training in error management.^18^ These findings suggest that, despite efforts to provide learning opportunities for managing errors in clinical practice, current initiatives may be insufficient to adequately equip junior doctors with the necessary skills for effective error management.

Neither the ACGME framework nor the CanMEDS roles describe errors and management of errors as directly as the UK’s framework Good Medical Practice.^15^^,^^20^^,^^21^ They describe the legal aspects of complaints and adverse events and how doctors should strive to improve patient safety. This prompts the question of whether the ability to manage errors is predominantly embedded within the practices of medical professionals and, consequently, acquired through the hidden curriculum.^22^^,^^23^ The objective of this study is to examine residents' perspectives on the training they receive in managing errors. It addresses the research question: how do residents perceive and experience their training in managing errors in clinical practice?

## Methods

### Design

The study used a qualitative and explorative design based on seven virtual focus groups,^24^ as focus groups encourage exploration of workplace culture by prompting group members to express "typically unspoken normative assumptions".^25^

### Study setting

In Denmark, the context for this study, both undergraduate and postgraduate medical education are based on the CanMEDs framework.^21^^,^^26^ The initial year of postgraduate medical education is considered foundational. During this year, doctors undergo two six-month rotations, typically in emergency medicine and general practice. Following this, there is a one-year introductory phase in specific specialties before doctors choose their specialized training paths. The education system is competency-based and features a relatively egalitarian hierarchy compared to other nations. An adverse events reporting system was introduced in Denmark in 2003 whereby healthcare workers and patients could report adverse events and errors anonymously.^5^ Some medical schools have incorporated the error management in their curricula. In the blueprints for specialty training in Denmark, managing errors primarily focuses on rules and regulations concerning patients’ rights, handling patient complaints and the adverse event reporting system, as well as identifying factors that are conducive to and unfavorable for learning.^27^

### Participants

The participants in the seven focus groups comprised 22 residents at different levels of education (from first to fourth year) in 11 various specialties. Demographics and distribution across specialty and educational level are presented in Table 1.

**Table 1:** Demographics of junior doctors (N=22) participating in the study

Variable		Medical^1^	Surgical^2^	Technical^3^	Total number
Gender					
	Men	4	1	1	6
	Women	11	3	2	16
Education					
	Foundation Year	9	-	-	9
	Introductory Year	2	-	2	4
	Main specialist training	4	4	1	9
Total number		15	4	3	22

Table 2 shows the distribution of participants in the study regarding gender, educational level and specialty. 1Family medicine, Internal medicine, Community medicine, Psychiatry, Pulmonary medicine, Oncology and Pediatrics; 2Gynecology and Obstetrics, Otology; 3Genetics, Pharmacology.

All focus groups were homogenous regarding educational level, but heterogeneous regarding medical specialty. Recruitment of participants continued until data saturation was obtained.^24^

The residents received written information about the project and were assured confidentiality. They gave verbal and video-recorded consent. The study was exempted from ethics approval according to the Act on Research Ethics Review of Health Research Projects. The local research committee was notified and the Danish Data Protection agency approved the study (No. 2022-0367531, 3113).

The residents were anonymized with a personal identification code: the first letter indicates educational level (K=Foundation year; I=Introductory year; H=Main Specialist training), followed by the number of the interview, while the second letter identifies each participant. Specialties are clustered into three groups: medical, surgical and technical (e.g. Genetics, Pharmacology) to ensure anonymity.

### Data collection

We used purposeful sampling to ensure a comprehensive range of perspectives, and participants were selected from residents across varying levels of experience, spanning from first to fourth year and from various specialties. The focus groups were conducted using Zoom’s video-conferencing system by the third and the last authors. It has been argued that online and virtual formats prove efficacious in convening individuals dispersed across diverse geographical locations.^28^ The interviews followed a semi-structured interview guide, including the themes: 1) experiences with medical errors, 2) communication and education relating to medical errors, 3) disclosure, and 4) management and social support. The focus groups were recorded and transcribed verbatim. The recordings were stored in a protected server in accordance with the security regulations provided by Danish Data Protection agency.

This study is part of a larger research project that explored how doctors experience intercollegial communication, training and education about errors. Results on intercollegial communication have been published previously,^19^ while results regarding training and education on managing errors are presented in this article.

### Data analysis

The data were analysed using reflexive thematic analysis.^29^ See Table 2 for a description of the steps in the analysis and our strategies for ensuring trustworthiness. A diverse team of researchers spanning communication, education, law, and medicine conducted the analysis; the various researcher perspectives strengthened the analysis.^30^

**Table 2:** Overview of steps in the data analysis

Step	Process of the study
1. Familiarization with data	All authors read the interviews and reflected on initial patterns to obtain a sense of the entirety of the junior doctors’ experiences of medical errors
2. Generating initial codes	To ensure reliability, all authors coded interviews individually (three researchers per interview) before comparing the findings and agreeing on final themes and subthemes. This ensured researcher triangulation. Disagreements were discussed, the material was re-read and consensus was achieved.
3. Searching for themes	All authors discussed patterns and defined themes and subthemes describing the data in relation to the study objective. Three overarching themes were developed: 1) How junior doctors conceptualize and experience talk and error management; 2) Junior doctors’ reaction to errors, and 3) How junior doctors describe their education in managing errors. The first two themes are reported elsewhere.^19^
4. Reviewing themes	The first and last authors specifically analysed all passages in the interviews concerning education on error management.
5. Defining themes	The final themes and subthemes were discussed and agreed on and validated by all authors. Disagreements were discussed and consensus was achieved.
6. Writing analysis	The first author wrote the final analysis. This was checked and commented on by all authors.

The resulting codes and themes with citations are shown in Table 3.

## Results

Data analysis resulted in the development of three themes that described how the residents perceived and experienced their education and training in error management: 1) formal education, 2) culture and clinical context, and 3) the need for more training. In the following, each theme will be unfolded with illustrative quotes that indicate the origin of the data.

### Formal education

Formal education in error management was understood as time that was specifically set aside for training in managing errors either via course-based or clinically-based education. The participants’ general impression was that errors and error management were neither prominent in their clinical training nor strongly present in the undergraduate or postgraduate curriculum, or as expressed in the following quote:

“I can’t even remember having received formalised teaching or instruction on managing errors [in medical school].” (H3G, female, main specialist training, technical**)**

The few participants who reported that they had been taught about errors at university mainly described having heard about the legal aspects of errors, but they had limited memory of specific teaching or instructions on how to manage errors in clinical practice.

“I do not really think that we were taught [about managing errors] at university, but we heard about the legal complaint system.” (K9F, female, foundation year, medical)

Formal teaching on error management during specialist training mainly took place as part of mandatory courses or the departments’ introduction programs and primarily involved the legal aspects of patients’ complaints or how to report to the adverse events system. This lack also applied to managing one’s own emotional responses:

“I do not think that (I have learned) what I should do myself if I make a mistake, or how I could work with my own reactions.” (K3E, female, foundation year, medical)

Furthermore, lack of focus on managing medical errors related to communication with patients as well as more generally, as witnessed in the following quote:

“I cannot remember ever receiving any kind of training in how to communicate about mistakes or how to manage them more generally. I have the impression that it might be different now, but I think, for the generation of doctors like mine, that it has been one of those soft values, one of those seven other doctor roles, which are not exactly being a medical expert, which is assumed to be known. Like it comes naturally. Once you've read all these books about diseases, everything else just follows naturally. Uh... (...) Yes, that's how I've experienced the expectation.” (H4B, female, main specialist training, medical)

**Table 3:** Examples of themes and sub-themes from the analysis supplied with citations

Category	Theme	Sub-theme	Examples	Citation
Formal Education				
	Courses	Undergraduate and postgraduate courses	Laws, rules and regulations, including patient complaints	“Well, we learned about the law and rules, and what to say to the patient... And there was a little about where to get help, e.g., from the union. But nothing about self-help and what you could do yourself” (K6E)
			No lessons	“I really do not think we were taught [about this] at university except about complaints – not so much about what you should do yourself” (K9F)
		Postgraduate courses/ teaching	Mandatory courses	“We discussed the adverse events reporting system on the mandatory course” (H2A)
			Departmental teaching	“It proved to be the adverse events reporting system that was on the agenda [in the departmental teaching program]. We did not get to discuss anything else regarding errors” (K5E)
			No teaching	“I do not remember being taught how to communicate about errors or how to manage them” (H4B)
Culture and clinical training				
	Formal forums where errors are discussed	Morning conference	Senior talks about errors	“I remember one of my older colleagues at a conference saying: ‘I have made this error….’” (K1D)
		Staff meeting	Adverse events are brought up	“Where I work, the theme “adverse events” is a fixed point on the agenda of the monthly staff meetings” (H1A)
	Informal forum where errors are discussed	Junior doctors’ office		“The error that had happened - well, that was something I happened to hear about in the junior doctors’ office” (H4B)
		On mandatory courses (during the breaks)		“[You hear about it] when you talk with your colleagues during mandatory courses – I guess it is a way of getting it out in the air”(K1D)
		Talk in the corners		“In the emergency department, there was a lot of talk [about errors], but not out in the open” (K6E)
	Apprenticeship learning in the clinic	See / hear / learn how others do		“I learned about it from the other doctors in the clinical department… they told about how they manage errors” (K3D)
		Guidance		“I wish somebody had had a talk with me along the lines: ‘Let’s have a look at this’ [error]” (K4E)
	Context / culture	Zero-fault culture		“Generally, I think that you are taught not to make errors” (I1C)
		Taboo	We hide in the corners – there is no openness	“Well, I think that generally we have a culture where you try to hide it” (H3A) “I guess it is kind of taboo, probably because doctors have a tendency to think that they never fail” (K9F)
		We learn from errors		“The best places I have worked were where you talked about errors and where it was OK to make errors” (K7F)
		Ending / Follow-up	How did it end?	“I was not part of [the error] but, for one reason or another I did not hear how the situation was resolved, but maybe nobody knew that I needed this?” (H4B)
			Feedback on reporting adverse events	“We got a list every month of all the adverse events that had been reported – what was done and so forth” (H3A) “Where is the learning from adverse events? When we report a systemic cause of error, it is really seldom we get any feedback on what was done – so the learning potential is lost” (H2A)
	Demands	Formalised teaching in error management, including how to disclose errors	How I am supposed to react towards:The patient, Myself, My colleagues,The organization	“I do not think I am trained to just go and tell or talk to somebody about [an error]” (H3A) “We are not trained in what to do if we make an error” (I2C) “I do not think that (I have learned) what to do myself if I make an error or how I could work with my own reactions” (K3E) “I do think that we should have more training on how to manage errors personally. You cannot look up how to overcome a bad conscience or that feeling of pain in the stomach” (K4E)
		Supervisor competence	Supervisors not trained	“I do not know ifsupervisors are trained in how to handle trainees who make an error, but I do think that would be a good idea” (K4E)
Need for more training				
	Suggestions to improve junior docors’ training			“Part of our responsibility as junior doctors is to train and talk about them [errors and error management]”

The perception was that learning such skills was not part of the official curriculum but could be learned by itself and thus was not taken as seriously as the ‘medical expert’ competencies. This inattention contrasts with all the residents remembering having made an error or having heard about doctors who had made one.

The residents described how errors were discussed at morning conferences – not as a planned teaching activity, but more as an opportunity for learning about how to manage a medical problem. Some had experiences of departments that regularly scheduled discussions about medical errors and adverse events at staff meetings. This was highly valued and perceived as ‘really instructive and helpful’. As one resident described it:

“Well, in the practice where I am, we have staff meetings once a month, and we have a fixed agenda item called “adverse events” where we talk about and internally discuss if something has been done wrong. And I think it is very educational. We also discuss, well, should we report it as an adverse event, or what should we do. I think it is very educational; and especially if you create an environment where people dare to say something, it can also be educational for others. So I think it is a very good way to do it.” (H3A, female, main specialist training, medical)

**Culture and informal clinical training**

In the clinical setting, informal training in error management varied from department to department. The residents reported that they occasionally met a supervisor who willingly described their own experiences of errors or taught them about error management. One junior doctor described how previously, when he was less experienced, he had difficulty finding out how to disclose to a patient an error in medical treatment that he had made. He asked a senior doctor for help; the senior doctor took responsibility for the issue and demonstrated how to disclose errors to patients:

“But I found it extremely difficult back then, so I allied myself with an older colleague, and I asked if he would help me, and he said, ‘You know what, I'll take care of it and tell him.’ So, there was an older colleague who stepped in. I joined him during the rounds, and I stood in the corner, like a little naughty schoolboy (laughs). But I remember that he said it exactly as I would have formulated it myself, and I think, actually, that's what I learned a lot from. He said it very candidly: There is something we are really sorry about, and fortunately, it happens rarely,’ I remember he said. ‘But a mistake has been made, in your case’.” (H6B, male, main specialist training, medical)

While such situations were experienced as useful and powerful learning situations, they were rare. Most junior doctors reported that errors only seemed to be discussed in informal settings like the junior doctors’ office or during breaks in mandatory courses in specialist training:

“When I was employed in the emergency department, there was a lot of talk, but nothing really official. At least not at our level. We had a patient who came in after a suicide attempt and during hospitalization almost succeeded again because no one had reacted to the fact that this patient shouldn't be left alone in the room. And there was a bit of gossip afterwards like: 'Oh, that was really bad' and ‘Think if…‘and 'Phew, have you heard…?'. But I can’t remember that we discussed it officially.” (K4E, female, foundation year, medical)

The participants generally described the impression that errors were not tolerated, and that the medical profession was characterized by a zero-fault culture where errors were not talked about or part of the curriculum of becoming a specialist, as expressed in the following:

“I think that you are generally taught not to make errors.” (I1C, female, introduction year, technical)

This was a recurrent experience, and all participants found it problematic learning how to manage errors, as error management seemed to be done in secret.

However, departments did differ regarding degrees of openness, from departments where errors seemed taboo, to departments where errors and managing errors were addressed openly:

“The best places I have been were where you talked about errors and where it was OK to make errors.” (K7F, male, foundation year, medical)

This cultural difference in openness was important because it was part of the departments’ learning environment and made a difference in how learning about managing errors took place. Several junior doctors mentioned the paradox that one should learn from errors but that after errors had occurred, they generally did not get to hear what happened to the patient or if there were any reprisals affecting the personnel or the department involved.

### Need for more training

All participants expressed the need for more focus and training on medical error management. Some wished that they had received more formal undergraduate training:

“Well, it would have been so cool if you were better prepared to manage errors.” (K3E, female, foundation year, medical)

In addition, more formal as well as informal focus on managing errors during residency training was requested. The contrast between the reality of facing errors and the lack of knowledge of how to manage them was seen as causing negative emotions about being a doctor. They found that the lack of training could have negative consequences, and some expressed having seen severe outcomes among their peers.

The residents experienced the need for training on how to disclose errors to patients, how to communicate with colleagues about errors, and how to handle their own reactions to errors. However, they questioned whether supervisors were equipped with the knowledge needed to teach about errors, as articulated in this quote:

“I do not know if supervisors are trained in how to handle trainees who make an error, but I do think it would be a good idea. Because, I haven't made that many mistakes, but I made one in general practice, and my tutor's handling was just, uh, not ideal. I thought afterwards, 'Wow'. If he had handled it a bit more seriously, I think I would have had a little more peace of mind. (…) I would have preferred that someone had sat down with me and said: 'Okay, let's just take a look at it. What exactly happened? And what consequences has it had? Has it had any consequences at all, or is it just something inside your head?', which it most likely was.” (K4E, female, foundation year, medical)

Thus, concrete dialogue that focused on the resident’s perspective and needs was suggested as beneficial. It was expressed that if supervisors had the knowledge and skills, this could positively impact junior doctors' general training in managing errors.

A few of the residents made the point that they were partly responsible for this, and a first year resident expressed that junior doctors themselves needed to train to become better teachers and role models for future generations:

“It's important to focus on how we create a culture where we talk more about mistakes. As junior doctors, it's part of our responsibility to discuss these errors. Because things won't change if we don't make an effort. It can be difficult to address the matter upwards, but perhaps we can encourage more discussion downwards, especially as we move forward in our specialist training.” (K9F, female, foundation year, medical)

## Discussion

This study shows that the residents considered their education in error management to be insufficient. They emphasized lack of important knowledge and training in disclosure and managing reactions to errors, aspects they considered poorly represented in the undergraduate curriculum and as only occurring sporadically in specialist training. They did learn about the legal aspects of errors, complaints and adverse events, and they were instructed in how to report to the adverse events system. However, they felt unprepared when they or their colleagues were involved in an error, and. they emphasized the need for practical training in error management to facilitate collective learning among colleagues and to enhance their ability to communicate effectively with patients. Furthermore, the junior doctors described the contradiction between the rule of conduct that you should ‘learn from your mistakes’, when at the same time they found that talking about errors seemed to be taboo in some departments. This highlighted a notable deficiency in training across multiple dimensions.

Our study showed that training in disclosure only occurs haphazardly through doctors’ clinical education. This is problematic as other studies have described how important it is to patients that doctors can manage adverse events and errors professionally and with empathy.^11^^,^^16^ When errors occur, patients want full information, an apology and reassurances that healthcare workers learn from the errors and undertake initiatives to prevent similar errors in the future.^11^^,^^16^ Factors affecting physicians’ willingness to disclose errors, has suggested to include facilitating factors like responsibility towards patients, profession, self and community, and barriers like attitudes, uncertainty, helplessness, fear and anxiety.^31^ These aspects would be relevant to include in specialty training on error managing. Recurrently, studies have pointed out that disclosure should be part of doctors’ education.^8^^,^^10^^,^^11^^,^^14^^,^^16^^,^^17^

The participants could not remember specific undergraduate training in managing medical errors and expressed that they lacked this to feel prepared. However, in Denmark, some medical schools do have short courses on the topic. This discrepancy could indicate that more focus on the topic in the pre-graduate setting is needed. It might also indicate that the training is placed too early on, and that it is forgotten before it is experienced in practice. If this explanation is true, then training should maybe be placed in the clinical setting, i.e., during the latter part of undergraduate training and in postgraduate training.

The importance of transparency regarding errors in clinical practice has been stressed previously.^9^^,^^17^^,^^32^ Our study shows that the culture around errors still differs significantly between departments where in some, errors and talk about errors are almost taboo, while in others, adverse events and errors are discussed openly and are fixed items on the agenda. Even though an adverse events reporting system has existed for more than 20 years, there are still departments where errors are not discussed systematically.^19^

This type of variation is documented in other studies, which show that most graduate medical education programs do not formally address diagnostic error prevention and reporting.^33^ Consequently, the desired shift from a “blame and shame” culture to one focused on learning from errors has yet to be fully realized.^3^^,^^34^ Research has demonstrated that structured initiatives can foster a more open culture and provide systematic learning opportunities.^3^^,^^35^ Such efforts should be integrated into broader postgraduate training programs to ensure consistency in residents’ learning experiences. This is particularly important because the prevailing perception of medicine as upholding a 'zero-fault' culture renders errors unacceptable and may impede the adoption of a learning-based approach to error management.^19^^,^^34^

Several authors have described the severe emotional reactions that healthcare workers experience when an error occurs and the importance of preparing healthcare workers to manage both their own and their colleagues’ emotional responses.^3^^,^^6^^,^^8^^-^^11^ Similar to a recent study from Canada, our study shows that residents want to be prepared to manage both the legal aspects of and the emotional reactions to errors.^18^ Given that all doctors at some point in their working life will be involved in errors,^2^ it might be time to consider incorporating systematic teaching about disclosure, emotional reactions and support to colleagues in the curricula and training programs in specialist training. This would require a revision of the Danish interpretation of the seven roles of the physician, incorporating a theme like “When things go wrong,” described in Good Medical Practice in the UK. Such training should include training senior doctors too, as we believe that clinical departments are the appropriate and relevant setting for this training. Placing the responsibility on departments and general practice for training residents in error managing would, however, require as a first step more openness towards adverse events and errors.

### Limitations

This study explored residents’ perceptions of how knowledge of how to manage errors was included in specialist training and as such did not take into consideration that other healthcare providers and colleagues might have other viewpoints. There is a skewness in the distribution across both educational level and specialty due to a preponderance of doctors in the second half of the Foundation Year. This means that general medicine is overrepresented, since the majority of Danish doctors work in general practice in their second half-year. However, the stories and recollections of the residents' experiences extended beyond their current position and were not limited to general medicine. Some of the informants could not remember being taught about error management in medical school. Error management is a rather new topic in medical schools in Denmark, and the informants may have left medical school before it was implemented, potentially leading to a skewness in the picture of training in managing medical errors.

## Conclusion

This study shows that residents perceived education in error management as being insufficient in specialist training. According to the residents included in our study, disclosure is not an adequate part of specialist training, and neither are emotional reactions of both the first and second victims, support of second victims, and the effects on third victims. More emphasis on these aspects requires a revision of the Danish interpretation of ‘the seven roles of the physician’ and the blueprints for specialist training and should involve ‘training the trainers’ to ensure the needed training in the clinical setting where errors occur. However, it also would require a more open culture around medical errors.

### Acknowledgements

We wish to thank all the residents who shared their experiences regarding errors in health care. The study was financially supported by and registered in the Central Denmark Region. Reg. No: 2022-0367531.

### Conflict of Interest

The authors declare that they have no conflict of interest.
